# Evaluation of extreme sub-daily precipitation in high-resolution global climate model simulations

**DOI:** 10.1098/rsta.2019.0545

**Published:** 2021-04-19

**Authors:** Michael Wehner, Jiwoo Lee, Mark Risser, Paul Ullrich, Peter Gleckler, William D. Collins

**Affiliations:** ^1^ Lawrence Berkeley National Laboratory, Berkeley, CA, USA; ^2^ Lawrence Livermore National Laboratory, Livermore, CA, USA; ^3^ University of California at Davis, Davis, CA, USA; ^4^ University of California at Berkeley, Berkeley, CA, USA

**Keywords:** extreme precipitation, high-resolution global climate models

## Abstract

We examine the resolution dependence of errors in extreme sub-daily precipitation in available high-resolution climate models. We find that simulated extreme precipitation increases as horizontal resolution increases but that appropriately constructed model skill metrics do not significantly change. We find little evidence that simulated extreme winter or summer storm processes significantly improve with the resolution because the model performance changes identified are consistent with expectations from scale dependence arguments alone. We also discuss the implications of these scale-dependent limitations on the interpretation of simulated extreme precipitation.

This article is part of a discussion meeting issue ‘Intensification of short-duration rainfall extremes and implications for flash flood risks’.

## 1. Introduction

Extreme precipitation at sub-daily scales can have significant flooding impacts in both urban and rural environments. Climate change is expected to increase the risk of such impacts as the magnitude of short-term extreme precipitation will increase in many regions due to increases in available moisture and energy. Confidence in the projection of these future increases as well as the attribution of current changes, if any, requires that climate models both simulate observed sub-daily extreme precipitation statistics well and adequately represent the relevant physical processes causing severe storms. Climate models in recent coordinated international projects such as CMIP5 and CMIP6, the 5th and 6th generation of the Coupled Model Intercomparison Project [1,2], are typically configured at effective horizontal grid resolutions of 100 km or coarser. For dynamical reasons alone, many properties of the severe storms responsible for extreme precipitation cannot be resolved at these grid spacings, no matter how good the subgrid-scale physical parameterizations are [3,4].

Climate models at horizontal resolutions of approximately 20–50 km have been shown to improve upon this situation [5]. In particular, the stronger gradients in moisture and temperature enabled at higher resolutions permit reasonable simulation of tropical cyclone properties [6,7] and other severe storm statistics [8–13]. Advances in high-performance computing technologies have progressed to the point where a limited number of multi-decadal simulations of climate models at these finer resolutions can now be performed. The multi-tiered HighResMIP subproject of the CMIP6 is the first attempt to intercompare the simulated past climate and projected future climate change of such models [14]. The HighResMIP protocols specify that modelling groups perform simulations with both a coarse- and fine-resolution model configuration. In practice, the coarse grid configurations are generally the operational version of the model and the high-resolution configuration an experimental version with grid spacings of 50 km or finer. Hence, the physical parameterizations in the models are specified by the protocols to be the same across resolutions. However, for stability reasons, some groups may have had to make minor parameter value adjustments, including time-stepping controls. The HighResMIP protocols specify both fully coupled ocean-atmosphere model configurations as well as atmosphere only configurations forced by fixed surface ocean and sea-ice datasets. Simulations of the recent historical period from 1950 to 2014 and a near-future period from 2015 to 2050 under the high emissions scenarios of RCP8.5 or SSP85 are called for in both the coupled and atmospheric-only configurations.

To the extent observations permit it, some aspects of sub-daily simulated precipitation have been evaluated including the diurnal cycle [15]. More recently, irregular sub-daily fluctuations about the mean diurnal cycle or ‘intermittency’ have been shown to be underestimated by models, even after taking into account the observational ‘error bars’ implied by different space–time resolutions [16].

In this paper, we use standard practice model evaluation techniques [17,18] to analyse the quality of seasonal 3-hourly precipitation extremes produced by available HighResMIP models. Previous evaluations of simulated extreme precipitation have focused on daily or pentadal accumulations [19–23].

Model evaluation is only as good as the observational datasets used as a reference and quality observed sub-daily precipitation accumulations are even more limited than for daily accumulations [24]. Furthermore, as shown by both Chen & Knutson, [25] and Gervais *et al*. [26] for extreme daily precipitation and further explored in this paper for extreme sub-daily precipitation, the order of operations in calculating gridded observational metrics can affect their magnitude and the interpretation of model quality. In this first evaluation, we thus confine our analyses to the conterminous United States (CONUS) and the winter (DJF) and summer (JJA) seasons.

To date, six modelling groups have submitted both coarse- and fine-resolution 3-hourly precipitation data to the historical period atmosphere only (*highresSST-present*) experiment. Several of these groups have also submitted simulations to the fully coupled model simulations. Errors in simulated sea surface temperature can significantly affect the location and intensity of severe storms that would likely degrade the quality of simulated extreme precipitation statistics. Hence, in this study, we focus our model evaluation on the more complete *highresSST-present* experiment and defer analysis of the effect of ocean-atmosphere coupling on extreme precipitation. We also add a seventh model that is not part of the HighResMIP but was integrated under similar boundary conditions.

In §2, we describe the merged radar and station observational dataset used as an evaluation standard and briefly describe the climate models with available fine- and coarse-resolution 3-hourly precipitation datasets. We also describe the effect of the order of gridding and extrema on the construction of a model evaluation standard in that section. In §3, we present the model error metrics including bias maps for each model and summary Taylor diagrams [27]. In §4, we discuss these errors and offer some interpretation of how model resolution affects the simulation quality of extreme sub-daily precipitation. We further discuss the limitations of simulated extreme precipitation and provide some context supplied by the expectations provided by the model evaluation standards. In §5, we summarize our principal conclusions about the effect of refined horizontal resolution on simulated sub-daily precipitation quality.

## 2. Methods, observations and models

Recognizing that the nature and magnitude of extreme storms in the mid and high latitudes is strongly seasonally dependent, we focus on the winter and summer seasonal extremes rather than on annual extremes. While long-period return values of seasonal maxima would be relevant for impacts, we focus only on the average winter and summer maxima as uncertainties from the short observational record in fitted extreme value distributions would be large, even with non-stationary statistical models [23]. However, we note that a previous model evaluation of average annual daily maximum precipitation and associated long-period return values [23] found that although model performance degrades as rarity increases, the patterns of errors are similar.

Long records of observed sub-daily precipitation data are a scarce resource and are available only over limited land regions from weather stations and/or radar. Sampling limitations currently make satellite-based products unsuitable reference data for our analysis. The HadISD [28] is an available multi-variate station dataset but precipitation is not one of the variables subjected to stringent quality control. The Global Sub-Daily Rainfall Dataset (GSDR), part of the INTENSE project [29], is the first real attempt to collect and quality control station-based sub-daily precipitation. Long, spatial dense records are mostly confined to the United States and some Western European countries. However, this dataset is not yet publicly available.

Operational weather radar provides a remote sensing alternative to ground-based observations. The National Centers for Environmental Prediction (NCEP) Environmental Modeling Center (EMC) has provided a merged ground-based and radar-derived hourly precipitation dataset from approximately 3000 weather stations and the 159 Doppler radars of the Next-Generation Radar (NEXRAD) on an approximately 4 km polar stereographic grid spanning the CONUS region [30] and is available at https://data.eol.ucar.edu/dataset/21.087. Most HighResMIP modelling groups provide 3-hourly precipitation accumulations so these hourly observations are similarly accumulated on the original stereographic grid from the raw downloaded data as the first step. We next used the data over the period June 1997 to February 2020 to calculate estimates of the average seasonal maximum of 3-hourly precipitation accumulation in two ways discussed below. Note that there are substantial missing data throughout this period, especially prior to 2002.

As mentioned above, the order of operations in the construction of the reference observations can introduce false estimation of model biases that are likely to be larger for sub-daily than for daily extreme precipitation model performance metrics [25,26]. Following the arguments of Chen & Knutson [25], the most reasonable interpretation of model-simulated precipitation is as an areal average. Thus, the observational extreme precipitation product most similar to model output is obtained by first gridding the raw high-frequency data to the model grid then calculating block extrema, usually annually or seasonally. As precipitation is by definition a moisture flux, this procedure should be made conservative. In this paper, we refer to such model evaluation results as the ‘native grid’ results since the observational extremes are calculated on each model's native grid. In this case, a different reference set must be calculated for each model further adding to the complexity of the evaluation process.

**Figure 1.**
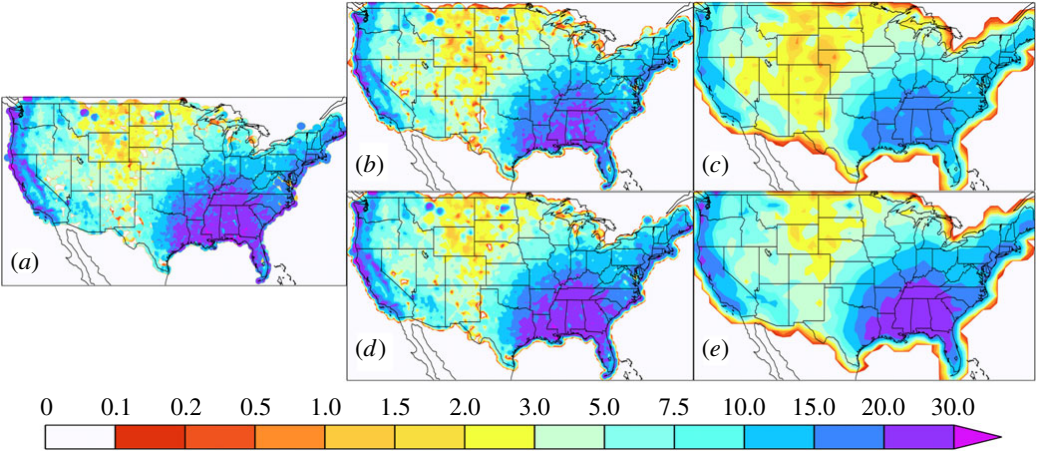
Average DJF maximum 3 h precipitation accumulation. (*a*) Maximum values calculated on the original 4 km polar stereographic mesh and regridded to a 4 km latitude-longitude mesh. (*b*) Maximum values obtained by first regridding daily precipitation to a 25 km mesh. (*c*) Maximum values obtained by first regridding daily precipitation to a 100 km mesh. (*d*) Maximum values obtained by regridding 4 km maxima to a 25 km mesh. (*e*) Maximum values obtained by regridding 4 km maxima to a 100 km mesh. (Coloured areas over coastal ocean regions are an artefact of the plotting scheme and should be ignored.) (Online version in colour.)

**Figure 2.**
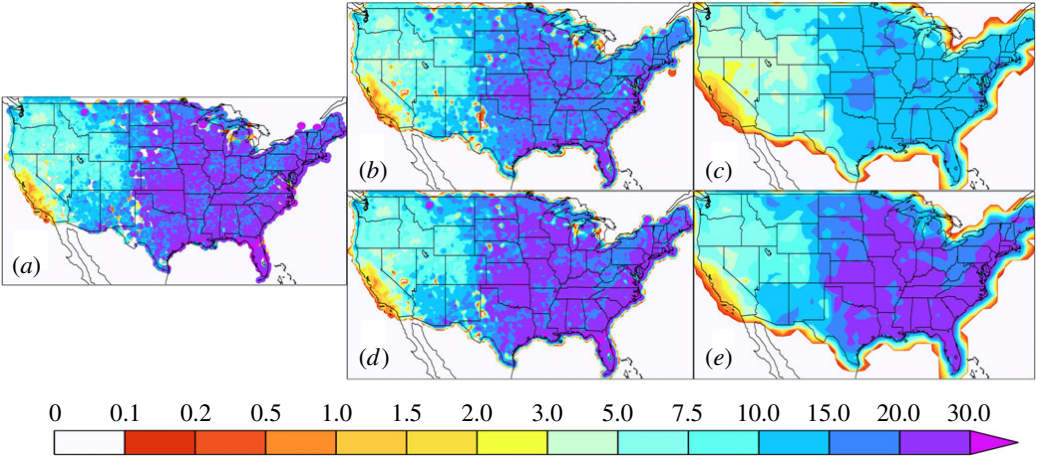
Average JJA maximum 3 h precipitation accumulation. (*a*) Maximum values calculated on the original 4 km polar stereographic mesh and regridded to a 4 km latitude-longitude mesh. (*b*) Maximum values obtained by first regridding daily precipitation to a 25 km mesh. (*c*) Maximum values obtained by first regridding daily precipitation to a 100 km mesh. (*d*) Maximum values obtained by regridding 4 km maxima to a 25 km mesh. (*e*) Maximum values obtained by regridding 4 km maxima to a 100 km mesh. (Online version in colour.)

However, this order of operation is not always practical, especially for sub-daily extremes due to the high computational cost of regridding and/or the availability of the high-frequency observational data itself. For instance, high-frequency station data may not be made available by the owners but block maxima or other extreme value indices are provided. In fact, this is the case for the daily extrema contained in the HadEX3 global land dataset [31] where the stations’ extrema are gridded rather than the stations' daily values themselves. In this paper, we refer to such model evaluation results as the ‘non-native grid’ since the observational extremes are not calculated on the models' native grid but are calculated either at individual stations or on a different grid. For precipitation, it is generally unlikely that the extrema at different locations within the same grid cell occur at the same time. Hence, observational estimates of gridded station extrema are generally larger than the extrema of gridded high-frequency station data. This order of operations bias also extends to the case where the observations are on a much finer grid than the models, as is the case here with the NCEP-EMC hybrid radar station product. Figures 1 and 2 show the observed average winter (figure 1*a*) and summer (figure 2*a*) maximum 3 h precipitation accumulations calculated on the original 4 km polar stereographic grid but regridded to a 4 km latitude-longitude for plotting purposes. Also shown in figure 1 are ‘native grid’ results at 25 km (figure 1*b*, figure 2*b*) and 100 km (figure 1*c*, figure 2*c*) and the ‘non-native grid’ results at 25 km (figure 1*d*, figure 2*d*) and 100 km (figure 1*e*, figure 2*e*). Clearly, the non-native regridding shown in the bottom rows result in values close to the original 4 km resolution. However, as Gervais *et al*., [26] and Chen & Knutson [25] point out, the smaller values produced by the native mesh regridding shown in the top rows are what the models should be expected to produce. Electronic supplementary material, figure S1 shows the per cent differences between the native and non-native gridding results further revealing that the effect of the order of operations is larger for lower resolutions than higher resolutions. In the next section, we show the effect of this order of regridding operations on model evaluation. Herein, we use the conservative and consistent TempestRemap package for regridding operations [32,33].

The CNRM-CM6-1 models were developed at the Centre National de Recherches Meteorologiques and the Centre European de Recherche et de Formation Avancee en Calcul Scientifique in Toulouse, France [34]. The EC-Earth3P models were developed by a consortium of European universities and laboratories (http://www.ec-earth.org) from Belgium, Denmark, Finland, Germany, Ireland, Italy, The Netherlands, Norway, Portugal, Spain, Sweden and the United Kingdom [14] and is based on the European Centre for Medium Range Forecasting IFS seasonal forecasting system [10]. The HadGEM3-GC3.1 is the current version of the United Kingdom's MetOffice Unified Model [35], and results were supplied at three resolutions. The IPSL-CM6A models were developed at the Institut Pierre Simon Laplace in Paris, France [36]. The MRI-AGCM3-2 models were developed at the Max Planck Institute for Meteorology in Hamburg, Germany [37]. The NICAM16 models are based on non-hydrostatic equations and were developed at multiple institutions in Yokohama, Tokyo and Tsukuba, Japan [38]. Additionally, we also evaluate the Community Atmospheric Model (CAM5.1), developed at the National Center for Atmospheric Research in Boulder, Colorado, United States [5,39]. While this model was not submitted to the HighResMIP subproject, it was integrated under similar boundary conditions for the period evaluated. The model names and their provided latitude and longitude dimensions are listed in table 1. However, models' true native grids may not be based on a latitude-longitude coordinate system and submitted data is regridded according to CMIP6 protocols in such cases. Interested readers are directed to the cited model documentation.

**Table 1.** Model resolution (column 2) and the number of realizations used in evaluation (column 3). Taylor's modified skill over the CONUS region for average maximum DJF and JJA 3-hourly precipitation using sub-daily observations regridded to the models' resolutions is shown in columns 5 and 6. High-resolution model versions are shown in italic font.

model	latitude × longitude	# of realizations	DJF skill	JJA skill
CAM5-1-1degree	128 × 256	49	0.75	0.54
*CAM5-1-2-025degree*	*360 × 720*	*5*	*0.79*	*0.60*
CNRM-CM6-1	256 × 512	1	0.67	0.65
*CNRM-CM6-1-HR*	*512 × 1024*	*1*	*0.76*	*0.68*
EC-Earth3P	144 × 192	3	0.73	0.68
*EC-Earth3P-HR*	*324 × 432*	*3*	*0.74*	*0.69*
HadGEM3-GC31-LM	143 × 144	2	0.68	0.53
HadGEM3-GC31-MM	361 × 512	2	0.74	0.52
*HadGEM3-GC31-HM*	*768 × 1024*	*3*	*0.80*	*0.54*
IPSL-CM6A-LR	320 × 640	1	0.70	0.52
*IPSL-CM6A-ATM-HR*	*960 × 1920*	*1*	*0.73*	*0.60*
MRI-AGCM3-2-H	320 × 640	1	0.82	0.57
*MRI-AGCM3-2-S*	*640 × 1280*	*1*	*0.82*	*0.56*
NICAM16-7S	192 × 288	1	0.77	0.46
*NICAM16-8S*	*768 × 1152*	*1*	*0.74*	*0.32*

The *highresSST-present* simulations nominally end in 2014 although some models end in 2015. For these simulations, we average the seasonal maxima over the 20-year period 1995 to 2014. The CAM5.1 model data is available only from 1996 to 2015, so we average over that 20-year period instead. While these periods are not identical to the observed period used here, the length of the period is about the same when accounting for the missing observations. While any anthropogenic trend in extreme precipitation from 2014 to 2020 is negligible, we admit that some differences due to natural modes of sea surface temperature variability might not be. However, Risser *et al*. [40] find that percentage of variance in extreme daily precipitation over the CONUS region explained by these modes is smaller than might be expected. Some models provide multiple realizations (table 1) and in these cases, the seasonal maxima are further averaged over realizations. The brevity of the reference observational period and the unavailability of multiple realizations from most of the HighResMIP models raises the question of the effect of internal variability on the uncertainty in model evaluation metrics. Using the 49 realizations of the low-resolution CAM5-1-1degree model, we have shown in the electronic supplementary material figure S4 that for a 20-year reference period uncertainty, in the magnitude of seasonal extreme precipitation errors for individual realizations, can be a significant fraction of the ensemble mean error. On the other hand, uncertainty in the error patterns from individual realizations is comparably low.

## 3. Model errors

**Figure 3.**
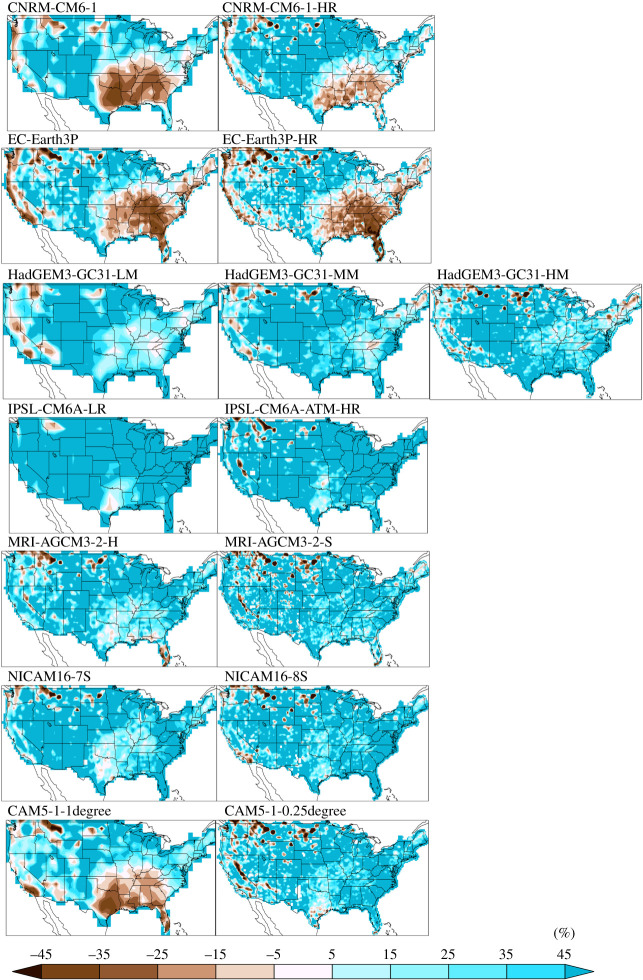
Per cent native grid error in simulated average DJF maximum 3 h precipitation accumulation. Models are arranged low to high horizontal resolution from left to right. (Online version in colour.)

Figure 3 shows the per cent error in simulated average DJF maximum 3 h precipitation accumulation using the native grid observations. Consistent with the expectation shown in the top row of figure 1, the high-resolution models produce larger maximum values than their lower resolution counterparts. Although there is little commonality between errors across the modelling groups, the patterns of native grid errors are remarkably similar across resolution within an individual modelling group. Electronic supplementary material, figure S2 shows the per cent error in simulated average DJF maximum 3 h precipitation accumulation using the non-native grid observations and highlights the importance of the order of operations in constructing the reference maxima. The native grid errors reveal that even the coarse models do not systematically underestimate extreme precipitation, contradicting a commonly held view. In fact, three of the models are generally too wet in the winter while the others are too dry in the wetter southeast and Pacific coast regions. Non-native grid model errors are very different to native grid errors since the non-native reference values, shown in the bottom row of figure 1, are so much larger than the native reference values of the top row of figure 1. The patterns of non-native grid model errors in the electronic supplementary material, figure S2 are much less similar across resolutions than the native grid model errors in figure 3 and can even be of opposite sign as summarized in the electronic supplementary material, table S1. This difference in error pattern can lead to very different and potentially incorrect conclusions about the sign of model bias as well as the effect of horizontal resolution on simulated extreme winter precipitation quality.

**Figure 4.**
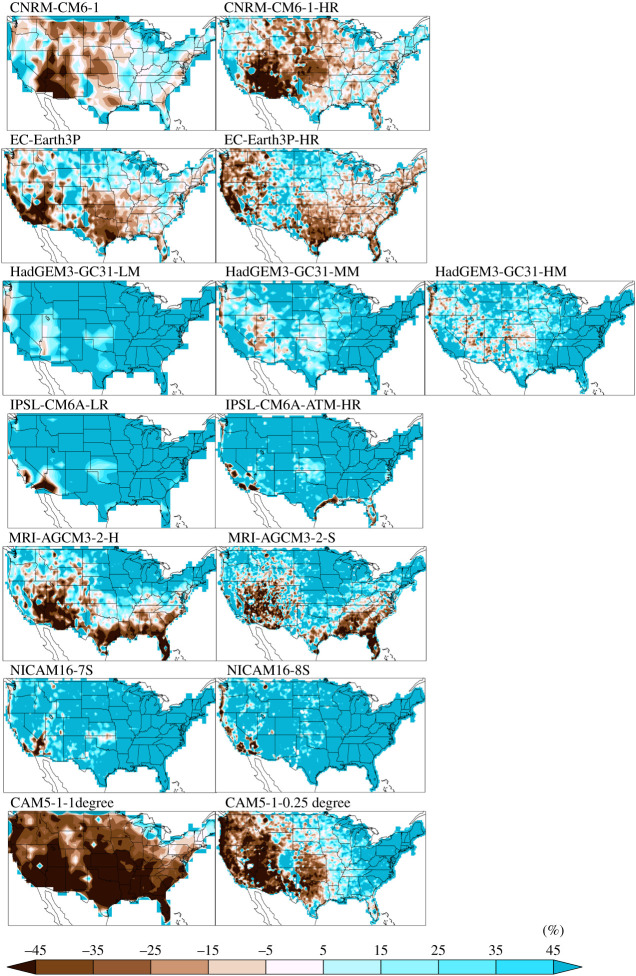
Per cent native grid error in simulated average JJA maximum 3 h precipitation accumulation. Models are arranged low to high horizontal resolution from left to right. (Online version in colour.)

Figure 4 shows the per cent error in simulated average JJA maximum 3 h precipitation accumulation using the native grid observations. Summer errors are generally considerably larger than winter errors at any resolution but bear little resemblance to them. Error patterns are again very different across models but for most models similar across resolutions. Three of the HighResMIP models are generally wetter than the observed summer extremes while the others are mixed without any significant reductions in native grid errors as resolution increases. The notable exception to similarity across resolutions is the CAM5.1 model in the southeastern US in both seasons. Electronic supplementary material, figure S3 and table S1 show that the non-native grid errors in summer would lead to generally incorrect conclusions about the magnitude and in many regions the sign of model bias.

**Figure 5.**
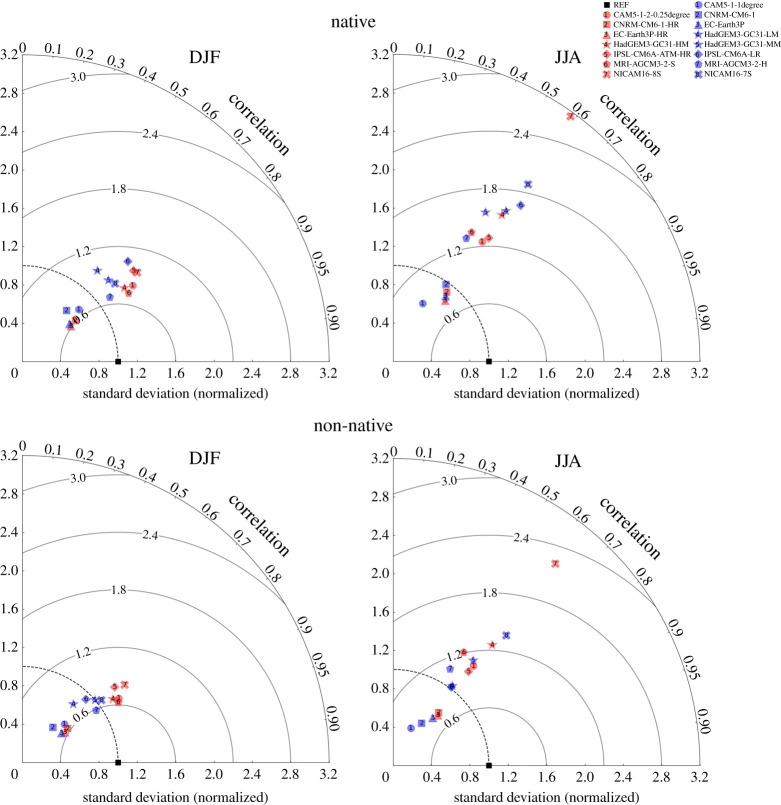
Taylor diagram of average DJF (*a*,*c*) and JJA (*b*,*d*) maximum simulated 3 h precipitation accumulation from native (*a*,*b*) and non-native (*c*,*d*) grid errors. Blue symbols are low-resolution models. Red symbols are high-resolution models. Symbol shapes are the same for models from the same modelling group. The concentric semicircles are isolines of normalized root mean square error. The dashed circle represents a normalized standard deviation of unity. (Online version in colour.)

We use Taylor diagrams to compare differences in selected centred statistics. Figure 5 shows Taylor diagrams of the pattern correlation and normalized spatial standard deviation of the simulated seasonal maximum 3 h precipitation accumulation for winter (left column) and summer (right column) using both the native (top row) and non-native (bottom row) grid observations. Traditionally, Taylor diagrams show the different model results computed on a common grid, but here centred statistics are calculated on each individual model's grid to be consistent with the resolution-dependent bias statistics. To facilitate comparison, we normalize the standard deviation of each model result by the corresponding value of the observed extremes. Consistent with the similarity in DJF native grid error structures across resolution shown in figure 3, there is little difference in the locations of symbols for the high (red) and low (blue) resolution simulations of a given modelling group shown in the upper left of figure 5. Despite the dissimilar winter error patterns across models shown in figure 3, points in the Taylor diagram are clustered in the angular dimension with centred pattern correlations between 0.6 and 0.8. However, there is considerable spread in the normalized spatial standard deviation indicating that the range of maximum values varies significantly across the CONUS region across models. Using the native grid observations, normalized Root Mean Square Error (RMSE) ranges from 0.6 to about 1.0 in the winter. Taylor's modified skill [41] in winter using the native grid reference, shown in table 1, ranges from 0.67 to 0.82 with generally small differences between simulations from the same modelling group. The equation for Taylor's modified skill is shown in the supplement.

The Taylor diagram of JJA native grid errors (upper right of figure 5) also shows a tight cluster around pattern correlation values between 0.5 and 0.6 (the angular dimension of the diagram) but a much larger spread in the radial dimension indicating a wider spatial dynamic range across modelling groups. As in winter, there is little difference in the placement of symbols across simulations from a given model group. Summer normalized RMSE is larger than winter ranging from about 0.9 to 2.5 and Taylor's modified skill (table 1) ranges from 0.32 to 0.69. Differences between simulations from the same modelling group again are small with the exception of the outlying NICAM16 models which exhibit exceptionally large simulated JJA 3 h maximum precipitation accumulations.

Perhaps surprisingly, considering the difference in model biases between the native and non-native grid standards, there is little corresponding difference in the Taylor diagrams. Pattern correlation metrics are essentially the same and any differences come from the normalized standard deviation.

## 4. Discussion

Part of the motivation for increasing climate models' horizontal resolution to a few tens of kilometres is to more realistically simulate some aspects of the severe storms responsible for extreme precipitation. And indeed, simulated seasonal maximum sub-daily precipitation accumulations increase with refined computational grids. The extrema based on appropriately coarsened sub-daily observations provide us the appropriate standard reference for model evaluation (upper panels of figures 1 and 2). Based on that standard, we find little improvement with grid refinement in simulated 3 h extreme precipitation accumulations when held to that expectation, at least over the CONUS region (figures 3–5).

**Figure 6.**
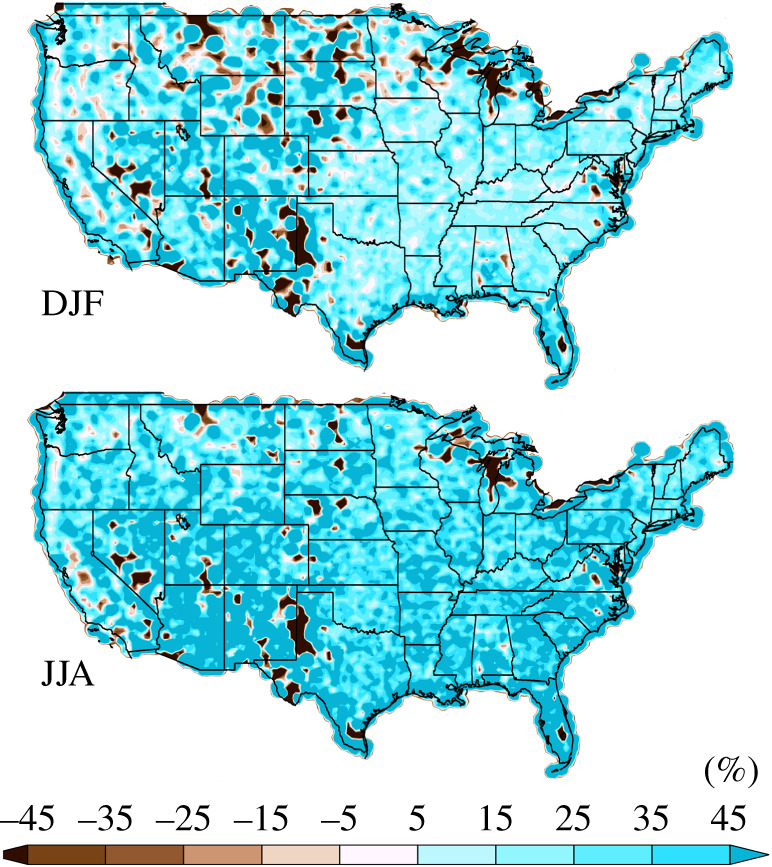
The expected per cent change in simulated average seasonal maximum 3 h precipitation accumulation for a change in horizontal resolution from approximately 100 km to approximately 25 km. (Online version in colour.)

The difference in the resolution-dependent standards in the upper panels of figures 1 and 2 provide an expectation of the increase in simulated extreme precipitation with resolution. Figure 6 shows the expected per cent change in simulated average seasonal maximum 3 h precipitation accumulation for a change in model horizontal resolution from approximately 100 km to approximately 25 km. In this figure, the approximately 100 km standard was conservatively remapped to approximately 25 km and is used in the denominator. This expectation, based on simple areal reduction arguments, is mostly of an increase. Decreases are mostly localized and confined to dry regions in areas of high orography. The shortness of record, combined with high variability in these regions is the most likely explanation for these decreases, rather than deficiency in the areal reduction argument.

We find that high-resolution models generally exhibit similar patterns in per cent errors to their low-resolution counterparts. The similarity in sub-daily precipitation errors across resolution suggests that large-scale circulation errors are not affected much by resolution. It also suggests that the locations and frequency of winter and summer extreme storms resulting from the simulated large-scale circulation are also not greatly affected by resolution although that aspect of the HighResMIP simulations has not yet been evaluated. While the magnitude of extreme storms is substantially larger and hence more realistic at high resolution, the ‘native grid’ method of defining an extreme precipitation standard accounts for this and little resolution dependence can be robustly identified in per cent error magnitude when models are evaluated against that standard. Further evidence that resolution has little effect on extreme precipitation beyond what is expected by figures 1 and 2 is provided by the normalized error metrics of the Taylor diagrams (figure 5). The distance between points representing models of different resolution from the same modelling groups is small and both normalized RMSE and Taylor's modified skill (table 1) exhibit only minor improvements at high resolution. In fact, skill values of extreme 3 h precipitation accumulations over the CONUS region for the HighResMIP models are quite similar to global land skill values for extreme daily precipitation accumulations for the CMIP5 and CMIP6 models [23]. Indeed, as extreme daily precipitation errors are highly correlated to mean precipitation errors [23], it is not surprising that sub-daily errors would be closely related to daily errors.

There is no systematic error pattern across all modelling groups, for all but one pair of models with an ancestral relationship. The CNRM-CM6-1 models and EC-Earth3P models both descend from versions of the ECMWF IFS atmospheric model and have very similar winter error patterns although differ in the summer. The other models, except CAM5.1, are largely biased high in the winter but mixed in the sign of summer errors. Averaged over the CONUS region, all models, both low and high resolution are biased high in the winter (electronic supplementary material, table S1).

Model evaluation is only as good as the reference data available, and for sub-daily precipitation, there are many obstacles to constructing them from *in situ* and remote observations for sub-daily precipitation. While the NCEP-EMC hybrid station and radar dataset is relatively short at about 20 years, the differences between it and the model simulations are likely much larger than natural variability, even for extreme sub-daily precipitation accumulations. Other datasets covering larger regions and longer time periods constructed by gridding station extrema are now becoming available [29,42]. While gridded station extrema are useful for assessing the actual risk of extreme precipitation, they are inappropriate for evaluation of simulated extreme precipitation bias due to fundamental discrepancies in their definition relative to model representation. Climate model precipitation within a grid is best thought of as a moisture flux and is a conserved quantity in a well-constructed climate model. While available sub-daily *in situ* station or radar measurements within a computational grid cell may be sparse, placing them on a grid at the same frequency as sampled by the model and subsequently calculating maxima most closely resembles what models simulate. Clearly, gridded station maxima are a different quantity than the maxima of gridded high-frequency precipitation and have no conservative properties. This same statement holds true for remapping very finely gridded observational maxima to a coarser grid. This is most clear by recognizing that within a grid cell, not all locations will experience the maximum precipitation accumulation at the same time. Hence, the gridded maxima are always larger than the maxima of gridded high-frequency precipitation. This effect is exacerbated as grids coarsen as shown in figures 1 and 2 and electronic supplementary material, figure S1.

This inconsistency between gridded maxima and what climate models actually simulate presents a challenge to comprehensive model evaluation. However, as figure 5 shows, a standard based on gridded maxima does provide useful information about the patterns of errors. Normalized RMSE and Taylor's modified skill from such a standard are biased but not as much as might be expected from the biases in error magnitude. This behaviour will prove useful in a limited model evaluation over a larger fraction of the planet when observed gridded maxima products such as from the INTENSE project become available.

We must point out that gridded extrema are indeed useful for other purposes, if not for the evaluation of the magnitude of model bias. For if one requires the risk of extreme precipitation at a point, long-period return values calculated from some variant of gridded extrema, preferably borrowing strength using spatial statistics, is the most credible estimate [43] as spatial smoothing damps some of the sampling variability.

There are important ramifications for the interpretation of simulated extreme precipitation from the reduced expectations of the proper averaging technique shown in upper panels of figures 1 and 2. First and foremost, return values or periods as calculated from climate models are not to be interpreted as representing the probability at a point of a specified extreme value. Although beyond the scope of this paper, they may be ways to use the top and bottom rows of figures 1 and 2 to bias correct the grid effect. Whether these errors cancel when inferring changes in the future probability of extreme precipitation from simulated return periods differences [44] remains an open question.

If the models simulate extreme precipitation statistics at values close to those from station or radar data (i.e. the lower panels of figures 1 and 2), then they are actually biased high. Also in extreme event attribution studies (e.g. [45]), models are often queried about the probability of a rare event of a given observed magnitude. However, comparison of climate model precipitation return values to the station or radar values describing a rare event leads to an overestimation of event probability in an unbiased model, even if the observations are placed on the same grid.

Trends in average and extreme precipitation are usually presented either as absolute or per cent changes from a reference period. Maps of absolute changes tend to highlight wet areas. Because of these reduced expectations, absolute changes in extreme precipitation of a given return period obtained from climate models would also be low in an unbiased model, if that return period is to be interpreted as a probability at a given point or region. The magnitude of the reduced expectations from the high-frequency gridding is likely a function of the rarity of the extreme precipitation considered. This would also introduce biases in point-wise probability changes interpreted from simulated per cent changes in long-period return values, although the magnitude of these errors would depend on how strong a function of rarity the reduced expectations are.

Likewise, similar caveats should be recognized in formal Detection and Attribution (D&A) analyses of observed trends in extreme precipitation [46,47]. ‘Scaling factors’ are a ratio of the observed to simulated trends and are tested against zero to infer causality in many D&A approaches. If observations are based on gridded extrema (lower panels of figures 1 and 2) and the climate models are unbiased, lower bounds of scaling factors of absolute extreme precipitation trends would be overestimated, possibly leading to erroneous causal inference. We note that Min *et al*. [46] accounted for general model biases by converting precipitation to a probability index and mitigated this problem.

## 5. Conclusion

Increasing global atmospheric model horizontal resolution increases the magnitude of simulated extreme sub-daily precipitation. In that sense, resolution increases are an important step towards a more realistic estimation of their behaviour. However, the expected magnitude of simulated extreme precipitation from areal reduction arguments is a strong function of resolution and when held against this standard, we find improvements in simulation quality to be nominal. In principle, horizontal resolution increases should improve the representation of extreme storms, and in actual practice, they do with tropical cyclones being a well-studied case in point. Hence, the lack of substantial improvement in the quality of simulated extreme sub-daily precipitation is puzzling, at least in winter and summer. Model errors in summer are larger than in winter, suggesting that parameterization of cumulus convection plays a role in these errors. However, even at high resolutions, most of the models examined herein are significantly too wet in the winter, suggesting that moisture transport errors also play an important role.

## Data accessibility

All HighResMIP model output data is available via the Earth System Grid at https://esgf-node.llnl.gov/search/cmip6/

CAM5.1 model data is available via the C20C data portal at https://portal.nersc.gov/c20c/

The NCEP-EMC observed precipitation data is available at https://data.eol.ucar.edu/dataset/21.093.
